# Comparative Genomics Reveals Genetic Adaptations to Diving‐Associated Foraging in Anseriformes

**DOI:** 10.1002/ece3.73551

**Published:** 2026-04-20

**Authors:** Tian Xia, Lei Zhang, Yuchun Li, Shuhong Li, Xiaodong Gao, Shengyang Zhou, Jianqun Ding, Guolei Sun, Xibao Wang, Xiufeng Yang, Xiaoyang Wu, Honghai Zhang

**Affiliations:** ^1^ College of Life Sciences Qufu Normal University Qufu China; ^2^ Shandong Key Laboratory of Wetland Ecology and Biodiversity Conservation in the Lower Yellow River Qufu China; ^3^ Yantai Forest Resources Monitoring and Protection Service Center Yantai China; ^4^ Beijing Key Laboratory of Captive Wildlife Technologies, Beijing Zoo Beijing China

## Abstract

Diving behavior in waterfowl represents a remarkable physiological adaptation requiring extensive genetic modifications to cope with hypoxic and high‐pressure underwater environments. We analyzed 25 Anseriformes genomes, representing diverse foraging strategies, and identified 13,873 orthogroups with 7991 single‐copy orthogroups across all species. Comparative genomic analysis revealed significant positive selection signatures in diving waterfowl, with GO and KEGG enrichment analyses highlighting key functional adaptations in metabolic regulation, transmembrane and immune responses. Metabolic pathways showed enrichment in insulin secretion and peptide hormone regulation, while transport mechanisms dominated molecular functions, including ion channels and solute transporters essential for maintaining cellular homeostasis under hypoxic conditions. Neural adaptations were evident through enrichment of postsynaptic density components and neurotransmitter transport systems. We identified nine genes with shared amino acid substitutions across diving species, including critical genes *PLB1*, *PKD1*, and *GPR34*. These findings demonstrate convergent molecular evolution in diving waterfowl, revealing the genetic basis underlying successful aquatic foraging strategies.

## Graphical abstract

We conducted a comparative genomic analysis of 25 Anseriformes species with diverse foraging strategies to uncover the genetic basis of diving adaptation. Diving waterfowl exhibited signatures of positive selection and functional enrichment in metabolic regulation, transmembrane transport, and neural processes essential for hypoxia tolerance. Shared amino acid substitutions in key genes such as *PLB1*, *PKD1*, and *GPR34* indicate convergent molecular evolution supporting aquatic foraging behavior.

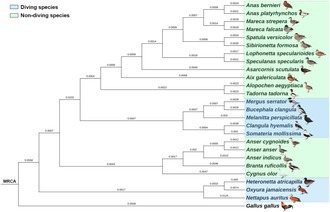

## 1 Introduction

Anseriformes (waterfowl) are widely recognized for their extensive distribution, occurring on all major continents and islands worldwide except for Antarctica (Kear 2005). Waterfowl, geese, and swans (Anseriformes: Anatidae) are valuable models for studying how morphology adapts to different foraging strategies and diets, as they not only share many physical traits suited to life on water but also display diverse feeding behaviors and food preferences (van der Leeuw et al. 2003). The main foraging strategies include: (1) grazing on terrestrial vegetation, as seen in many geese (Anserini); (2) surface feeding by dabbling or tipping up, typical of dabbling waterfowl (Anatini); and (3) diving to obtain food. Foraging tactics and diets differ among diving species, with diving waterfowl or pochards (Aythyini) performing shallower dives to consume aquatic plants and invertebrates, while mergansers (Mergini) dive deeper in pursuit of invertebrates and fish (Lisney et al. 2013). These diverse foraging strategies have been linked to anatomical traits such as the lacrimal/ectethmoid region (De Mendoza et al. 2020), body mass (Olsen 2015), and visual system specialization (Lisney et al. 2013).

Foraging innovations can either promote morphological diversity by creating new environmental interactions or constrain it through functional limitations imposed by specific foraging strategies (Eliason et al. 2020). Diving, as a trait dependent on highly specialized morphological adaptations (Butler and Jones 1997; Felice and O'Connor 2014), may represent a key innovation that opens new ecological opportunities. Prior research on diving birds has investigated how energy acquisition and foraging efficiency are optimized, often focusing on prey capability (Draulans 1982), temporal efficiency (Wilson 1988), and diet composition (Ball 1994). Halsey et al. further demonstrated that while feeding duration in diving birds typically correlates with dive duration (Halsey, Reed, et al. 2003), it is also influenced by additional ecological factors, including food density (Halsey, Woakes, and Butler 2003), prey selection time (Draulans 1982), and ingestion rate (Stephenson et al. 1986). Evolutionary origins of diving have been explored across multiple avian lineages, such as penguins (Cole et al. 2022), Charadriiformes (Smith and Clarke 2012), loons and grebes (Clifton et al. 2018), dippers (Smith et al. 2022), and kingfishers (Crandell et al. 2019). However, comparisons among distantly related diving taxa can make it difficult to distinguish genetic changes associated with diving foraging from signals shaped by broader ancestry and ecological variation. In this context, Anseriformes provide a more suitable evolutionary framework: within a single order, diving has evolved repeatedly alongside non‐diving relatives, enabling internal controls, while the order‐level diversity of diving strategies helps to capture variation in aquatic foraging ecology.

In this study, we analyzed Anseriformes species for which high‐quality genomes are available to comprehensively identify genes exhibiting evolutionary signatures in diving lineages. The species selected for this study include both diving and non‐diving Anseriformes, enabling comparative analyses that are phylogenetically informed and better reflect variation in diving foraging ecology. We aimed to uncover molecular adaptations associated with the evolution of diving foraging behavior. Through comparative genomics analyses, we detect genes showing significant positive selection in diving waterfowl and genes harboring convergent amino acid substitutions shared across diving lineages relative to non‐diving relatives. We further show that multiple genes and pathways involved in the regulation of diving‐associated foraging traits display distinct evolutionary patterns in these lineages. Collectively, our results provide molecular evidence consistent with convergent evolution underpinning successful aquatic foraging strategies in diving waterfowl.

## 2 Methods

### 2.1 Genome Collection and One‐to‐One Orthologs Identification

The whole genome sequences of 25 Anseriformes species and an outgroup ( *Gallus gallus* , GCF_016699485.2) were downloaded from the NCBI (National Center for Biotechnology Information, https://www.ncbi.nlm.nih.gov/datasets/genome) database (Table 1). To ensure the reliability of downstream comparative genomic analyses, we assessed the assembly quality of each genome using two complementary metrics: scaffold N50 and gene completeness (evaluated with BUSCO v5.4.7 against the aves_odb10 dataset). While the majority of genomes showed high completeness (BUSCO > 90%), *Melanitta perspicillata* and *Mergus serrator* exhibited slightly lower completeness (85.1% and 88.0%, respectively). Due to variable annotation quality across genomes, we employed a reference‐based approach using the well‐annotated mallard ( *Anas platyrhynchos* ) as a reference. We aligned all genomes to the reference using LAST (v.956; parameters: ‐m50 ‐E0.05 ‐C2) and MULTIZ (v.10.6), extracted coding sequences (CDS) from aligned regions (≥ 70% identity, ≥ 50% coverage), and translated them to protein sequences. We then employed OrthoFinder (v.2.4.0) (Emms and Kelly 2015) to identify high‐confidence “one‐to‐one” orthologous gene clusters across all Anseriformes species, utilizing the all‐against‐all DIAMOND (Buchfink et al. 2015) algorithm for orthology inference.

**TABLE 1:** Genomic data and foraging strategies of 25 Anseriformes species.

Scientific name	Foraging strategy	Assembly	Scaffold N50	BUSCO
*Aix galericulata*	Dabbling	ASM2463536v1	78.2 Mb	94.60%
*Anas bernieri*	Dabbling	ASM3216521v1	2.8 Mb	90.80%
*Anas platyrhynchos*	Dabbling	ZJU1.0	80.2 Mb	94.30%
*Asarcornis scutulata*	Dabbling	ASM1339847v1	76.3 Mb	94.30%
*Lophonetta specularioides*	Dabbling	ASM3216523v1	79 Mb	95.80%
*Mareca falcata*	Dabbling	ASM3838208v1	81 Mb	95.60%
*Mareca strepera*	Dabbling	ASM3558391v1	65.8 Mb	94.00%
*Sibirionetta formosa*	Dabbling	ASM3840621v1	5.6 Mb	94.60%
*Spatula versicolor*	Dabbling	ASM3838200v1	12.6 Mb	90.80%
*Speculanas specularis*	Dabbling	ASM3216525v1	66.4 Mb	95.40%
*Tadorna tadorna*	Dabbling	ASM4578464v1	78.1 Mb	95.90%
*Cygnus olor*	Dabbling	bCygOlo1.pri.v2	81.5 Mb	93.50%
*Bucephala clangula*	Diving	bBucCla1.1	23.4 Mb	94.20%
*Clangula hyemalis*	Diving	bClaHye2.1	79.7 Mb	94.10%
*Melanitta perspicillata*	Diving	ASM4578343v1	76.7 Mb	91.40%
*Mergus serrator*	Diving	ASM4578214v1	80.4 Mb	92.40%
*Nettapus auritus*	Diving	BPBGC_Naur_1.0	62.1 kb	85.10%
*Somateria mollissima*	Diving	bSomMol1.h1.1	111.7 kb	88.00%
*Heteronetta atricapilla*	Diving	BPBGC_Hatr_1.0	26 Mb	94.30%
*Oxyura jamaicensis*	Diving	BPBGC_Ojam_1.0	36.1 Mb	94.60%
*Alopochen aegyptiaca*	Walking	ASM4578331v1	76.1 Mb	91.40%
*Anser anser*	Walking	bAnsAns1.hap1.1	77.6 Mb	92.60%
*Anser cygnoides*	Walking	Taihu_goose_T2T_genome	76 Mb	92.90%
*Anser indicus*	Walking	ASM2558372v1	80 Mb	94.10%
*Branta ruficollis*	Walking	ASM4578450v1	6.6 Mb	93.60%

### 2.2 Phylogenetic Analysis

All genes were aligned using the MUSCLE program (Edgar 2004), and the resulting alignment was refined by removing gaps and highly variable regions through Gblocks (v 0.91) (Castresana 2000). We employed RAxML software (version 8.2.12) (Stamatakis 2014) to generate maximum‐likelihood phylogenetic trees using the parameter configuration “GTRGAMMA ‐f a ‐x 12345 ‐N 100 ‐p 12345 ‐o GalGal” with tree topology confidence evaluated through 1000 bootstrap replicates.

### 2.3 Selective Pressure Test for Diving Birds

To assess the patterns of selective pressure in diving birds, we estimated the nonsynonymous (dN) to synonymous (dS) substitution rate ratio (ω) using the codeml program employed in PAML (v 4.10.7) (Yang 2007). Eight diving waterfowl (*Bucephala clangula, Clangula hyemalis, Heteronetta atricapilla, Melanitta perspicillata, Mergus serrator, Nettapus auritus, Oxyura jamaicensis*, and *Somateria mollissima* ) were designated as foreground branches, corresponding to the terminal branches leading to these eight diving species in the species phylogeny. These diving taxa are distributed across two distinct branches and do not form a monophyletic diving clade. The remaining Anseriform species were treated as background branches. Coding sequences of single‐copy orthologous genes were aligned using PRANK (Löytynoja and Goldman 2005), and any alignments that were less than 150 base pairs in length and contained gaps were excluded. Positively selected genes (PSGs) were then identified by comparing a branch‐site model (model = 2, NSsites = 2, fix_omega = 0), which allows for positive selection on specific sites along foreground branches, against a null model (model = 2, NSsites = 2, fix_omega = 1, omega = 1). We applied likelihood‐ratio tests and chi‐square analyses to evaluate statistical significance, with genes significantly deviating from the null model (FDR‐corrected *p* < 0.05) being classified as PSGs. Finally, Bayes empirical Bayes (BEB) analysis was performed under the alternative branch‐site model to detect codon sites under significant positive selection in the foreground lineages, defined as having a posterior probability > 0.95 (Yang et al. 2005).

### 2.4 Specific Amino Acid Changes and Protein Functional Annotation in Diving Waterfowl

The multiple sequence alignments of orthologous proteins of diving waterfowl and grazing and dabbling waterfowl allowed us to identify convergent amino acid residues. A Perl script was used to identify genes with amino acid changes unique to eight diving waterfowl compared to the 17 other species in our Anseriformes dataset. To investigate whether convergent specific amino acid sites are located within key functional domains of the corresponding proteins, we searched the SMART database (https://smart.embl.de/). The selected genes carrying convergent amino acid substitutions were retained for structural analysis. Protein three‐dimensional structures were predicted using the AlphaFold Server (https://alphafoldserver.com) with default parameters (Abramson et al. 2024). For each protein, five models were generated, and the highest‐ranked model (model_0) was selected for structural analysis. Structural alignment and visualization were performed using PyMOL (v3.11) (Delano 2002), and protein structure diagrams were generated.

## 3 Results

### 3.1 Single‐Copy Orthologous Genes and Phylogeny

**FIGURE 1:**
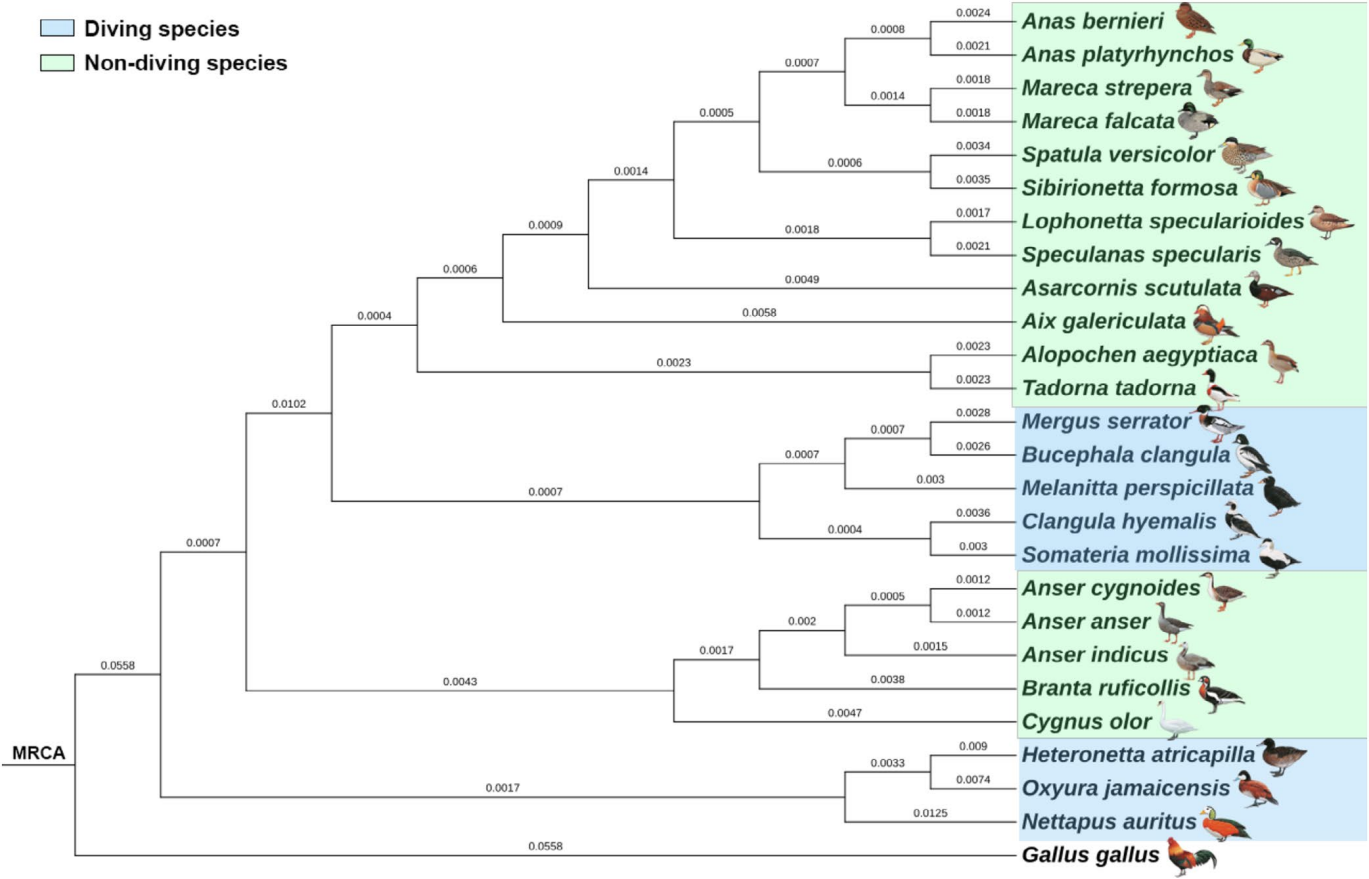
Phylogenetic tree of 25 Anseriformes species based on whole‐genome orthologues, with *Gallus gallus* as the outgroup. Blue branches indicate diving species (*n* = 8); green branches indicate non‐diving species (*n* = 17), including dabbling ducks and terrestrial geese.

Given the widely available whole‐genome data, we selected 25 genomes representing different foraging methods of the order Anseriformes. A total of 13,873 orthogroups were identified from all species using OrthoFinder, with 9502 orthogroups present in all species, of which 7991 consisted entirely of single‐copy orthogroups. These single‐copy orthologous genes were aligned and concatenated to construct a species phylogenetic tree, providing a robust framework for subsequent comparative genomic analyses. The phylogenetic tree illustrates the evolutionary relationships among diving and non‐diving waterfowl species. Diving species (highlighted in blue) are distributed across multiple clades, indicating that diving behavior likely evolved independently several times within the group (Figure 1).

### 3.2 Positive Selection in Diving Waterfowl

**FIGURE 2:**
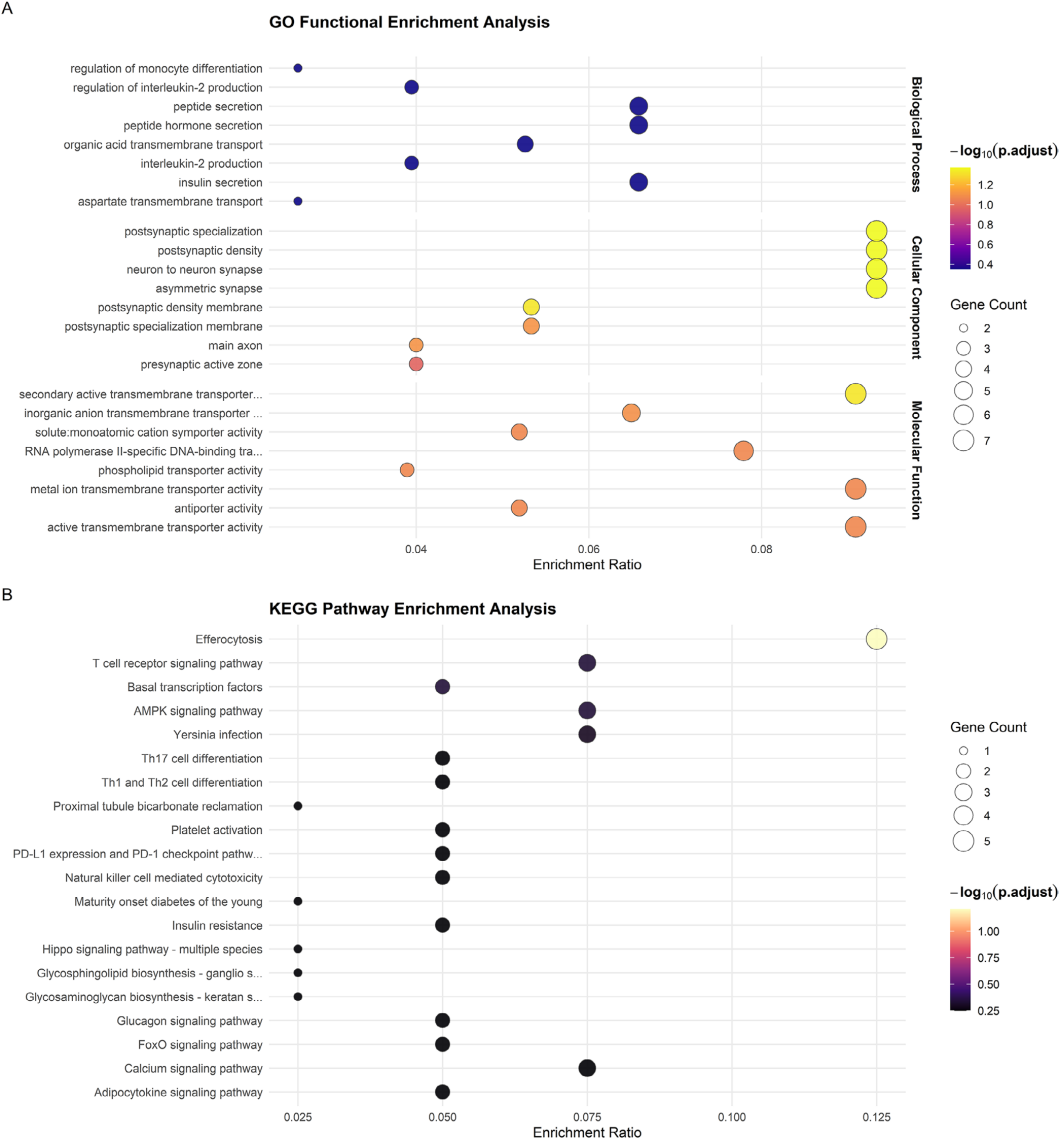
Functional enrichment of positively selected genes (PSGs). (A) Gene Ontology (GO) enrichment; (B) Kyoto Encyclopedia of Genes and Genomes (KEGG) pathway enrichment.

We identified genes with significant positive selection signatures (FDR < 0.05) in diving waterfowl. The gene list and detailed analysis results are presented in Table S1. We subsequently performed GO and KEGG functional enrichment analyses on these positively selected genes. Detailed enrichment results are presented in Table S2. Based on the GO enrichment analysis, the top 30 most significantly enriched terms (Figure 2A, Table S3) revealed key functional categories broadly related to metabolic regulation, transport mechanisms, neural signaling, and immune responses in diving birds.

#### 3.2.1 Metabolic and Endocrine Regulation

Both GO and KEGG analyses highlighted strong selection on metabolic and endocrine regulation. GO enrichment included processes such as insulin secretion, regulation of insulin secretion, peptide hormone secretion, and peptide secretion (Table S3). Complementarily, KEGG analysis identified significant enrichment in pathways related to the endocrine system, carbohydrate metabolism, lipid metabolism, amino acid metabolism, and nucleotide metabolism (Figure 2B, Table S4). These findings collectively suggest involvement in metabolic and hormonal adjustments, which may be relevant for energy management during diving.

#### 3.2.2 Transport Mechanisms and Cellular Homeostasis

**FIGURE 3:**
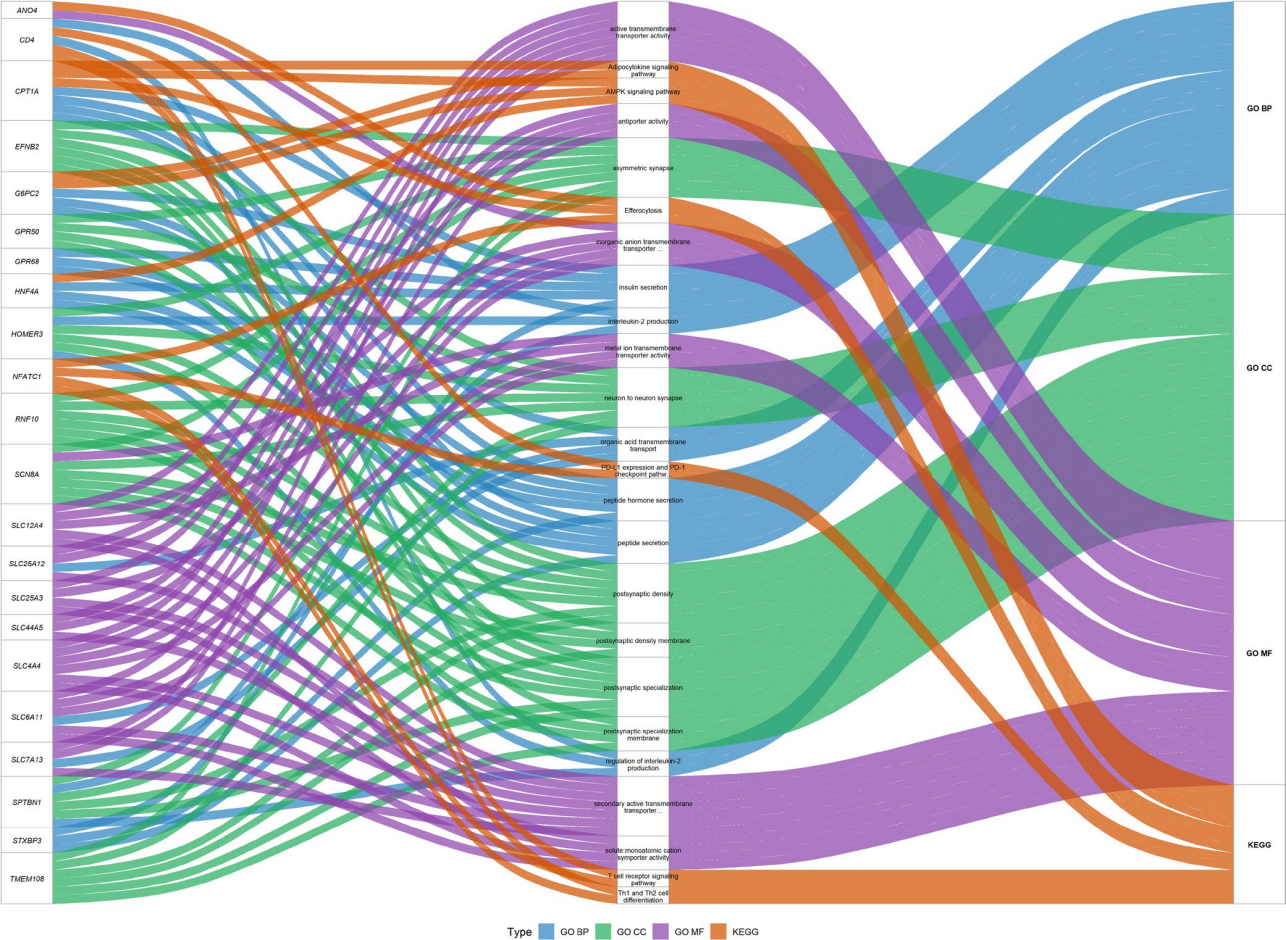
Sankey diagram of positively selected genes (PSGs) and functional pathways. Left column: PSGs; middle column: functional annotations (GO and KEGG terms); right column: pathway categories.

Transport mechanisms were extensively enriched, dominating GO molecular function categories. These included various transmembrane transporter activities (e.g., secondary active, inorganic anion, solute: monoatomic cation symporter, metal ion, active, phospholipid, symporter, and monoatomic anion transmembrane transporter activities). Related GO biological processes, such as aspartate transmembrane transport, organic acid transmembrane transport, and peptide transport, were also prominent (Table S3). Further supporting cellular homeostasis, KEGG analysis revealed enrichment in pathways related to transport and catabolism, alongside GO terms for glucose homeostasis and cellular response to oxidative stress (Figure 3). These observations suggest potential roles in maintaining cellular and systemic balance under the physiological demands of diving.

#### 3.2.3 Neural Function and Signaling

GO cellular component terms extensively featured synaptic structures, such as postsynaptic density, asymmetric synapse, postsynaptic specialization, neuron to neuron synapse, postsynaptic density membrane, main axon, presynaptic active zone, and early endosome (Figure 2C; Table S3). Consistently, KEGG analysis identified enrichment in pathways related to the nervous system and neurodegenerative disease pathways (Table S4). Furthermore, GO also showed enrichment in neurotrophin signaling pathways like nerve growth factor signaling pathway and calcium‐mediated signaling, possibly reflecting involvement of neural circuitry in rapid coordination and physiological responses.

#### 3.2.4 Immune Response and Transcriptional Control

Immune regulation was another key enriched domain. GO analysis included terms such as interleukin‐2 production, regulation of interleukin‐2 production, and regulation of monocyte differentiation (Table S3). KEGG analysis similarly highlighted the immune system, infectious disease, and immune disease pathways (Table S4), suggesting evolutionary refinement of immune defense mechanisms in diving waterfowl. Additionally, transcriptional control was represented by the GO term RNA polymerase II‐specific DNA‐binding transcription factor binding (Figure 3; Table S3), indicating adaptations in gene expression regulation.

#### 3.2.5 Broader Physiological Adaptations and Organ Systems

Beyond these major themes, enrichments also pointed to adaptations in respiratory processes (respiratory gaseous exchange by the respiratory system) and cardiovascular functions (heart contraction, cardiac muscle cell differentiation) in GO terms (Table S3). KEGG analysis further underscored comprehensive system‐level adaptations, including pathways for the circulatory system, digestive system, and excretory system, along with broader signal transduction, cellular process pathways (e.g., cell growth and death, cell motility), and environmental adaptation (Table S4).

### 3.3 Shared Amino Acid Changes

**FIGURE 4:**
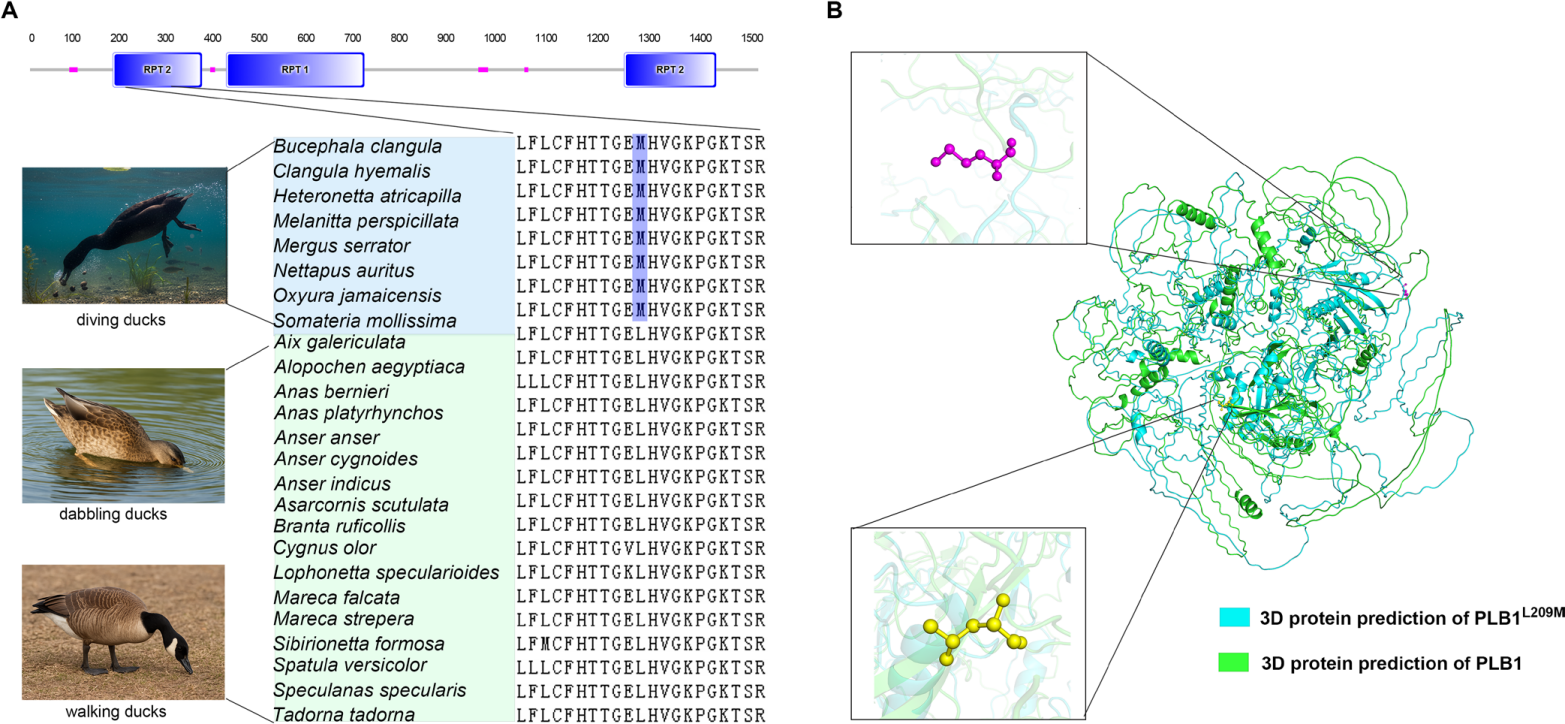
Convergent evolution and structural prediction of *PLB1* in diving Anseriformes. (A) Amino acid substitutions shared across eight diving species. (B) Predicted 3D structures of wild‐type and mutant protein variants.

The conserved amino acid substitutions observed in specific lineages may provide insights into the genetic mechanisms driving specific phenotypic adaptations. We established strict criteria where a site was defined as a shared amino acid substitution among diving waterfowl only if the amino acid was consistent across 17 non‐diving waterfowl and different from the amino acid in diving waterfowl. As a result, we identified 11 genes (*PLEKHG1, KIF11, PKD1, PLB1, SH3TC1, SLIT3, ARNT2, CRACDL, CHST9, GPR34, and THADA*) that underwent shared amino acid substitutions in eight diving waterfowl (Table 2). To investigate the potential functional significance of these convergent substitutions, we performed protein domain analyses for all 11 candidate genes. The results revealed that three genes (*PKD1*, *PLB1*, and *GPR34*) harbored amino acid substitutions located within annotated functional domains, suggesting possible impacts on protein function (Figure 4A).

**TABLE 2:** Genes with convergent amino acid substitutions in diving Anseriformes.

Gene	Position	Amino acid in non‐diving species	Amino acid in diving species
*ARNT2*	176	V	I
*CHST9*	246	G	S
*CRACDL*	492	P	S
*GPR34*	152	V	I
*KIF11*	975	V	M
*PKD1*	194	T	A
*PLB1*	209	L	M
*PLEKHG1*	1144	A	V
*SH3TC1*	1286	T	I
*SLIT3*	1067	H	R
*THADA*	144	S	P

### 3.4 Structural Modeling of *PKD1* , *PLB1*, and *GPR34*

To further assess the potential structural consequences of the convergent amino acid substitutions, we conducted protein structure modeling for *PKD1*, *PLB1*, and *GPR34*, in which the mutations were located within annotated functional domains. The predicted 3D structures were generated based on homologous templates, and the wild‐type and mutant models were compared. The results (Figure 4B, Figure S1) revealed that these substitutions induced distinct local conformational alterations near active or binding sites, which may affect protein stability and molecular interactions. Collectively, these findings suggest that the convergent substitutions in *PKD1*, *PLB1*, and *GPR34* may have functional significance and could contribute to the adaptive evolution of diving waterfowl.

## 4 Discussion

Diving, as a highly specialized foraging strategy, has long attracted research interest, from early physiological studies of diving behavior, respiration (Halsey and Butler 2006), diving physiology (Ponganis and Kooyman 2000), energetic costs (Butler 2000), and metabolic regulation (Butler 2004) to recent genomic investigations revealing convergent molecular changes in marine mammals and diving birds (Cole et al. 2022; Foote et al. 2015; Wang et al. 2023; Zhang et al. 2025; Xia et al. 2024). Our comparative genomic analysis of 25 Anseriformes species reveals that diving waterfowl have evolved extensive molecular adaptations. These findings demonstrate that diving behavior is underpinned by positive selection acting on functionally interconnected gene networks, rather than isolated genetic changes. Furthermore, we identified 11 genes with convergent amino acid substitutions specific to diving lineages, including *PKD1*, *PLB1*, and *GPR34*, which harbor mutations within conserved functional domains likely to affect protein activity. The multi‐system integration we document parallels convergent adaptations in marine mammals and penguins, yet extends previous work by revealing coordinated selection across physiological networks in a non‐marine avian lineage. This suggests that the genomic architecture of diving adaptation follows predictable evolutionary patterns across vertebrates, despite independent origins and varying ecological contexts.

### 4.1 Metabolic and Ionic Homeostasis: Convergent Solutions to Energetic Constraints

The enrichment of insulin secretion and peptide hormone regulation pathways aligns with documented selection on energy metabolism genes in diving vertebrates (Yuan et al. 2021; Cole et al. 2022; Khudyakov et al. 2015). We interpret these signatures as reflecting selection for metabolic flexibility—the capacity to rapidly switch between aerobic and anaerobic states during breath‐hold diving. This interpretation is supported by the co‐enrichment of transmembrane transport functions, suggesting that ionic homeostasis and metabolic regulation represent functionally coupled adaptations (Foote et al. 2015; Uribe et al. 2024; Vianna et al. 2020). Ecologically, these adaptations enable diving waterfowl to exploit benthic and pelagic food resources unavailable to surface‐feeding relatives, thereby expanding niche space. The repeated evolution of similar genomic solutions across phylogenetically distant divers suggests that energetic constraints imposed by breath‐hold diving impose strong selection pressures that override phylogenetic inertia.

### 4.2 Neural Efficiency and Immune Modulation: Balancing Performance and Protection Under Hypoxia

The observed selection on synaptic components and neurotransmitter transport systems is consistent with energy‐saving modifications in neural transmission documented in seal brains (Geßner et al. 2022). We propose that diving waterfowl have evolved optimized neurotransmission efficiency to reduce metabolic demand during oxygen‐limited conditions. This neural adaptation likely interacts synergistically with metabolic suppression—a hallmark of the dive response—enabling prolonged submergence. Concurrently, selection on immune regulatory pathways—including convergent substitutions in GPR34—reflects adaptation to the inflammatory consequences of repeated hypoxia‐reoxygenation cycles (Ming et al. 2019; Reyes‐Ramos et al. 2023). The oxidative stress generated during resurfacing poses a physiological trade‐off: while necessary for oxygen replenishment, reperfusion risks tissue damage via inflammatory cascades. The immune modifications we document suggest that diving waterfowl have evolved tolerance mechanisms that mitigate this trade‐off, balancing immediate survival needs against long‐term tissue integrity.

### 4.3 Cardiovascular Integration: Orchestrating Systemic Dive Responses

The enrichment of cardiac and respiratory pathways indicates tight coupling between circulatory and pulmonary systems, consistent with the dive response characterized by bradycardia and selective vasoconstriction (Hindle 2020; Blix and Folkow 1983). We posit that cardiovascular adaptations serve as the physiological orchestrator enabling metabolic, neural, and immune adaptations to function effectively during dives. The efficiency of cardiovascular control directly influences foraging profitability—the energy gained per dive relative to surface recovery time.

### 4.4 Candidate Genes and Molecular Mechanisms of Convergence

The 11 convergent amino acid substitutions we identified offer testable hypotheses regarding molecular mechanisms of diving adaptation (Table 2). Among these, *PKD1*, *PLB1*, and *GPR34* are particularly notable because each carries substitutions within annotated, conserved functional domains, and structural modeling predicts local conformational changes that are likely to affect protein activity or interactions.

The *PKD1* gene emerges as a compelling candidate for diving adaptation in waterfowl, primarily due to its conserved role in mechanosensation and pressure sensing. Substitutions within mechanosensory domains likely tune pressure sensing in vascular and epithelial tissues, given the established role of *PKD1–PKD2* complexes in mechanotransduction (Bezares‐Calderón et al. 2020, 2018; Nauli et al. 2003). During diving, waterfowl experience dramatic pressure changes that could stress cellular membranes and mechanosensory systems. The convergent substitutions we observe in *PKD1* may therefore reflect adaptive tuning of ciliary or endothelial mechanosensitivity and resilience, helping to maintain vascular and epithelial function under pressure fluctuations.

*PLB1* is involved in membrane dynamics under physiological stress, where domain‐specific mutations may modulate membrane phospholipid remodeling to facilitate rapid adjustments in membrane composition during diving cycles (Wright et al. 2007; Mukherjee et al. 2003). This environmentally responsive nature suggests that *PLB1* may play a role in maintaining membrane homeostasis under physiological stress. In diving waterfowl, the convergent amino acid substitutions identified within functional domains of *PLB1* may modulate its catalytic efficiency or membrane‐binding properties, thereby facilitating adaptive adjustments in membrane dynamics, lipid metabolism, and cellular resilience to hypoxia during repeated diving cycles.

*GPR34*, an orphan G‐protein‐coupled receptor implicated in immune regulation, contributes to the control of inflammatory responses and pathogen defense, and its deficiency causes dysregulated inflammation in model systems (Liebscher et al. 2011). Diving waterfowl experience a unique constellation of physiological stresses that directly impact immune function. Similarly, pressure changes associated with diving directly impact immune cell function, with increased hydrostatic pressure altering leukocyte responses and cellular metabolism (Thompson and Romano 2016). Previous research has shown that evolutionary modifications in immune‐related genes in penguins may have been crucial for their successful adaptation to underwater living conditions during their transition to aquatic life (Cole et al. 2022). The conserved‐domain substitutions and predicted conformational changes in *GPR34* observed here may therefore represent evolutionary modifications that tune immune signaling dynamics, balancing protection against pathogens with mitigation of dive‐induced inflammatory damage.

Among the remaining genes with shared amino acid substitutions, *ARNT2* and *THADA* are also noteworthy. A fixed amino acid substitution in ARNT2 aligns with emerging evidence of convergent molecular evolution in underwater‐tolerant taxa, as Yépez et al. documented similar adaptive signatures in aquatic mammals, where ARNT2 exhibited relaxed selective constraints and a site‐specific convergent substitution (Yépez et al. 2024). *ARNT2* functions as a critical component of the hypoxia‐inducible factor (HIF) pathway, forming heterodimers with HIF‐α subunits to regulate oxygen‐dependent gene expression. The substitutions we observed in this gene could modulate DNA‐binding affinity or complex stability, akin to the destabilizing HIF3α variant unique to cetaceans (Yépez et al. 2024). The HIF pathway, central to hypoxia tolerance, shows convergent evolution across aquatic mammals, with HIF‐related genes exhibiting positive selection in both marine and freshwater diving species (Allen and Vázquez‐Medina 2019). *THADA*, meanwhile, regulates energy metabolism and thyroid hormone pathways—critical for thermogenesis and basal metabolic rate elevation. In humans, *THADA* emerged as a key candidate for cold adaptation in indigenous Siberian populations, showing strong signals of positive selection (Cardona et al. 2014). In diving waterfowl, analogous selective pressures may drive *THADA* evolution. Prolonged submergence in cold aquatic environments necessitates efficient oxygen utilization, metabolic suppression during dives, and rapid thermoregulatory recovery.

### 4.5 Limitations and Recommendations

We acknowledge that positive selection signatures alone do not definitively assign functional roles to specific genes in diving behavior. Several alternative explanations should be considered. First, selection signals may reflect adaptation to correlated ecological traits—such as cold tolerance, aquatic foraging, or habitat use—rather than diving per se. Second, apparent convergence among diving species could be influenced by shared ancestry rather than independent adaptation, although our use of non‐diving relatives within Anseriformes as controls helps mitigate this concern. Third, while structural modeling suggests potential functional impacts of the identified amino acid substitutions, these predictions require empirical validation through functional assays. Future studies incorporating transcriptomic, proteomic, and knockout experiments will be essential to establish causal relationships between these genetic changes and diving adaptations. While our taxon sampling represents the most comprehensive genomic dataset for Anseriformes to date, certain diving lineages remain underrepresented. We note that future studies with expanded sampling will be essential to distinguish signals of convergent diving adaptation from lineage‐specific evolutionary trajectories and to enable more refined comparisons between shallow and deep divers.

## 5 Conclusion

Our comparative genomic analysis of 25 Anseriformes species reveals widespread signatures of positive selection in diving waterfowl, with functional enrichment in metabolic regulation, ion transport, neural signaling, immune modulation, and cardiovascular control—reflecting coordinated physiological strategies for tolerance to hypoxia and hydrostatic pressure. We identified 11 genes bearing shared amino acid substitutions across diving lineages, including *PKD1* (mechanosensation), *PLB1* (membrane remodeling), and *GPR34* (immune regulation), with structural modeling indicating potential functional impacts. These findings provide molecular evidence for convergent evolutionary solutions to the energetic, mechanical, and immune challenges imposed by diving. Together, they demonstrate that the evolution of diving behavior in Anseriformes involves integrated genomic adaptations paralleling those in other aquatic vertebrates, offering new insights into the genetic architecture of diving physiology and aquatic specialization.

## Author Contributions

**Tian Xia:** software (equal), visualization (equal), writing – original draft (lead), writing – review and editing (equal). **Lei Zhang:** software (equal), writing – review and editing (equal). **Yuchun Li:** resources (equal). **Shuhong Li:** resources (equal). **Xiaodong Gao:** formal analysis (equal). **Shengyang Zhou:** methodology (equal). **Jianqun Ding:** methodology (equal). **Guolei Sun:** formal analysis (equal). **Xibao Wang:** formal analysis (equal). **Xiufeng Yang:** resources (equal). **Xiaoyang Wu:** visualization (equal). **Honghai Zhang:** writing – review and editing (equal).

## Funding

This work was supported by the National Natural Science Foundation of China, 32270444, 32470448, 32570499; Natural Science Foundation of Shandong Province, ZR2023ZD47, ZR2025MS283.

## Conflicts of Interest

The authors declare no conflicts of interest.

## Supporting information


**Figure S1:** Structural prediction of convergent gene variants in diving Anseriformes. (A) PKD1 wild‐type and mutant protein structures. (B) GPR34 wild‐type and mutant protein structures.

**Table S1:** Positively selected genes (PSGs) in diving waterfowl (FDR < 0.05).

**Table S2:** Functional enrichment analysis results (GO and KEGG) for PSGs.

**Table S3:** The top 30 GO enrichment terms for PSGs.

**Table S4:** The top 15 KEGG enrichment pathways for PSGs.

## Data Availability

All data used in this study were obtained from the NCBI database, and the genome version information is provided in Table 1.
