# Supplementary figures and images for: Comparative Genomics Reveals Genetic Adaptations to Diving‐Associated Foraging in Anseriformes
Source: Ecol Evol. 2026 Apr 20;16(4):e73551. doi: 10.1002/ece3.73551 (PMC13095863; doi:10.1002/ece3.73551)

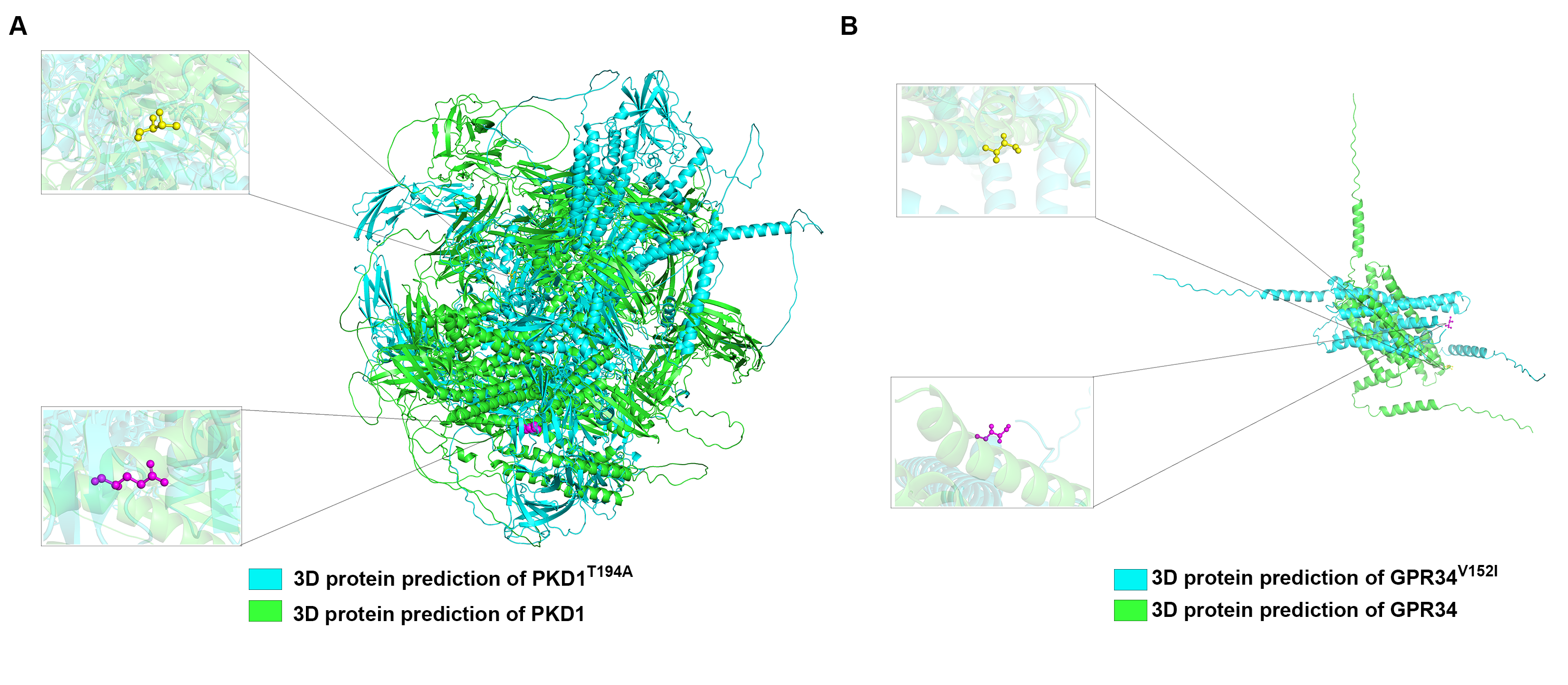

Supplement: Supplementary file 1 — Figure S1: Structural prediction of convergent gene variants in diving Anseriformes. (A) PKD1 wild‐type and mutant protein structures. (B) GPR34 wild‐type and mutant protein structures. [file ECE3-16-e73551-s004.png]
